# Season of birth and breast cancer risk in Sweden.

**DOI:** 10.1038/bjc.1994.346

**Published:** 1994-09

**Authors:** J. Yuen, A. Ekbom, D. Trichopoulos, C. C. Hsieh, H. O. Adami

**Affiliations:** Cancer Epidemiology Unit, Uppsala University Hospital, Sweden.

## Abstract

Recent research suggests that intrauterine exposures, perhaps factors that influence birth weight and other indicators of fetal growth, may affect future breast cancer risk. Because birth weight shows seasonal variation in Sweden, we assessed whether risk for breast cancer is associated with month of birth. The analyses included all 115,670 women, born between 1858 and 1968, who were reported to the Swedish Cancer Registry in 1958-89 as having breast cancer. Poisson regression models were used to examine the data. After adjustment for seasonality of number of live births in the population at risk, a significant seasonal pattern was identified for women born between 1880 and 1920. Women born in June had a 5% higher risk of breast cancer than those born in December. By contrast, there was no evidence of birth seasonality among 440,948 women with cancer at other sites. Exposures relevant to breast cancer risk later in life are unlikely to be related to month of birth. Thus, prenatal or early post-natal factors influence breast carcinogenesis, but the seasonal variation in these factors must have decreased over time.


Br. J. Cancer (1994), 70, 564-568 © Macmillan Press Ltd., 1994

### Season of birth and breast cancer risk in Sweden

J. Yuen[1], A. Ekbom[1,2], D. Trichopoulos[2], C.-C. Hsieh[2] & H.-O. Adami[1,2]

[1]*Cancer Epidemiology Unit, Uppsala University Hospital, S-751 85 Uppsala, Sweden;* [2]*Department of Epidemiology, Harvard School of Public Health, Boston, Massachusetts 02115, USA.*

**Summary** Recent research suggests that intrauterine exposures, perhaps factors that influence birth weight and other indicators of fetal growth, may affect future breast cancer risk. Because birth weight shows seasonal variation in Sweden, we assessed whether risk for breast cancer is associated with month of birth. The analyses included all 115,670 women, born between 1858 and 1968, who were reported to the Swedish Cancer Registry in 1958-89 as having breast cancer. Poisson regression models were used to examine the data. After adjustment for seasonality of number of live births in the population at risk, a significant seasonal pattern was identified for women born between 1880 and 1920. Women born in June had a 5% higher risk of breast cancer than those born in December. By contrast, there was no evidence of birth seasonality among 440,948 women with cancer at other sites. Exposures relevant to breast cancer risk later in life are unlikely to be related to month of birth. Thus, prenatal or early post-natal factors influence breast carcinogenesis, but the seasonal variation in these factors must have decreased over time.

Trichopoulos (1990) proposed that increased levels of pregnancy oestrogens – or other exposures *in utero* – may increase the future risk of breast cancer in daughters. This hypothesis was directly supported by two investigations (Ekbom *et al.*, 1992; Sandson *et al.*, 1992). Further, higher maternal age at birth (Thompson & Janerich, 1990), being first-born (Hsieh *et al.*, 1991) and being a twin (Hsieh *et al.*, 1992) are associated with increased breast cancer risk and also with increased levels of pregnancy oestrogens (Tamby-Raja & Ratnam, 1981; Bernstein *et al.*, 1986; Panagiotopoulou *et al.*, 1990). Finally, pathological studies of newborns have suggested prenatal influences on breast cancer risk (Anbazhagen & Gusterson, 1992; Anbazhagen *et al.*, 1992). Nevertheless, the hypothesis and the supporting evidence have been received by the scientific community with scepticism, aptly summarised in the statement that ' [the perinatal origin of breast cancer] stretch(es) biological credibility' (Miller, 1993).

We here present evidence that season of birth is a determinant of breast cancer risk. Without addressing the pregnancy oestrogen hypothesis, birth seasonality would suggest perinatal influences. Our examination was prompted by the realisation that the food supply in Sweden must have had a more seasonal variation at the beginning of the century than it does today, owing to poorer food preservation and handling methods.

### Patients and methods

Since 1 January 1958 all newly diagnosed malignant tumours in Sweden must be reported to the National Cancer Registry by both the physician who makes the diagnosis and the confirming pathologist or cytologist (The Cancer Registry 1960-92). At the time of this study the Registry was complete up to 31 December 1989. We used information on site of the tumour, the birth date portion of the patient's national registration number and the year of diagnosis. Through the Registry, we identified all females reported with a first invasive breast cancer (ICD-7, code 170) between 1958 and 1989, a total of 115,670 females. They were born from 1858 to 1968, 70% before 1920. We have also retrieved and computerised the number of live births (alive 24 h after delivery) by month from 1858 to 1968, recorded by Statistics Sweden and its predecessors.

Nine period groups were formed from the data based on decade of birth from pre-1880, 1880-1890 to 1950. Each period group was further stratified on the basis of month of birth, resulting in 108 observational cells (nine time periods by 12 months). Each patient was assigned to one of these cells based on her year and month of birth. Poisson regression models (Prener & Carstensen, 1990) were used to examine the data for seasonality using the log (number of births) in the multiplicative model.

Independent variables in the regression models included the decade effects (DEC), month as a categorical variable (MO) and month as a continuous variable (NMO). Month was also transformed by calculating sin (month $\times \pi/6$) and cos (month $\times \pi/6$) (SMO and CMO respectively) to explore the possibility of yearly cycles within the data. Likelihood ratio statistics were used to compare candidate models, and individual regression parameters were evaluated with 95% confidence intervals.

We also considered whether children born in particular months or seasons might have a higher probability of survival until adulthood. To assess this possibility we studied the birth seasonality among 466,336 women with cancer at sites other than the breast in the same database, again adjusting for seasonality of live births in the underlying population at risk.

### Results

Table I presents, by month of birth and by decade, the 115,670 women with breast cancer, the 440,948 women with cancer at sites other than the breast and the total number of live births from 1858 to 1968.

Month of birth was an important risk factor for breast cancer, but not for cancers at other sites. When month was added as a categorical variable to a model including decade only, the fit improved significantly for breast cancer (LRT = 37.72, 11 d.f.) but not for all other cancers combined (LRT = 14.71, 11 d.f.) (Table II). The natural logarithm of the relative risk for breast cancer increased to a peak in June, and then decreased (Table III). No pattern was present in the data for cancers at all other sites. These models fit the data poorly (Table II, model B). This poor fit was due to a strong interaction between month and decade for both breast cancer and cancer at other sites. The addition of an interaction term of month as a continuous variable with decade to a model with only decade was highly significant for breast cancer (LRT = 59.09, 9 d.f.) and for all other cancers (122.8, 9 d.f.), indicating that the linear effect of month varied with decade (Table II, model C). The effect of month (continuous) was positive for the earlier decades (increasing risk through the year), but steadily decreased and was negative for later decades. The interaction of month (categorical) with decade was not fitted, since this is a saturated model and would have left no estimate for error.

Correspondence: J. Yuen.
Received 8 November 1993; and in revised form 22 March 1994.

After adjusting for the confounding effects of month within each decade with the variable NMO, the risk of developing cancer still showed seasonal variation for breast cancer, but not for cancer at all other sites. The addition of a seasonal component common to all decades consisting of the variables SMO and CMO (Table II, model D) improved the fit of the model significantly for breast cancer (LRT = 27.38, 2 d.f.) but not for all other cancer sites (LRT = 2.292, 2 d.f.). For breast cancer, values of 0.0007 (0.004) and −0.0198 (0.004) were obtained for SMO and CMO respectively (log coefficient and standard error). These values indicate an increased risk for women born in the middle of the year compared with those born at the beginning or the end of the year. For cancers at all other sites, no significant seasonal variation was seen, and estimated coefficients for SMO and CMO were −0.0012 (0.0021) and 0.0034 (0.0021) respectively (log coefficient and standard error).

The possibility of an interaction (that the association between seasonality of birth and breast cancer risk might not be constant over calendar time) was examined by fitting a full model with seasonality components specific to each decade (Table II, model E). There was some evidence that the effect of month of birth on the risk of developing breast cancer varied from decade to decade, though no effect of month of birth was seen in the analyses for other cancer sites. For the breast cancer cases, the seasonal components were significant (LRT = 44.7, 18 d.f.) ($P<0.01$) but did not represent a significant improvement over the common cyclic component (LRT = 23.32, 16 d.f.). For all other cancer sites, a full model with decade-specific seasonal components was not significant (LRT = 15.25, 18 d.f.). Fitting the decade-specific cyclic components in succession to the breast cancer data yielded changes in −2 log likelihood of 0.85, 7.59, 4.52, 7.53, 9.86, 2.92, 1.10, 1.53 and 8.80 for each decade (from before 1880 to after 1950). Since each cyclic component consists of two values, one for SMO and one for CMO (both components must be added simultaneously to allow cycles to begin at any point within the year), these changes can be considered to be chi-square distributed with two degrees of freedom. This corresponds to probabilities of 0.35, 0.98, 0.90, 0.98, 0.99, 0.76, 0.42, 0.53 and 0.99 for the significance of the

**Table I** Total number of women with breast cancer and cancer at all other sites diagnosed in Sweden in 1958–89 among women born between 1858 and 1968; and total number of live births in Sweden from 1858 to 1968

| *Month of birth* | *Breast cancer* | *Other cancers* | *Live births* |
|---|---|---|---|
| January | 9,828 | 37,369 | 1,114,624 |
| February | 9,269 | 34,886 | 1,042,496 |
| March | 10,325 | 40,607 | 1,199,923 |
| April | 10,033 | 38,216 | 1,150,230 |
| May | 10,149 | 38,339 | 1,145,625 |
| June | 9,739 | 35,662 | 1,063,710 |
| July | 9,604 | 36,146 | 1,068,707 |
| August | 9,448 | 35,354 | 1,042,999 |
| September | 9,834 | 38,081 | 1,111,187 |
| October | 9,295 | 35,622 | 1,059,244 |
| November | 8,817 | 33,981 | 998,549 |
| December | 9,329 | 36,685 | 1,072,411 |
| 1858–1880 | 1,434 | 5,871 | 2,091,520 |
| 1880–1890 | 7,311 | 30,479 | 1,359,009 |
| 1890–1900 | 17,283 | 66,949 | 1,334,184 |
| 1900–1910 | 25,954 | 89,908 | 1,370,922 |
| 1910–1920 | 26,422 | 77,684 | 1,257,952 |
| 1920–1930 | 20,360 | 56,534 | 1,102,934 |
| 1930–1940 | 10,438 | 36,688 | 901,428 |
| 1940–1950 | 5,531 | 42,422 | 1,205,112 |
| 1950–1968 | 937 | 34,413 | 2,446,644 |
| Total | 115,670 | 440,948 | 13,069,705 |

**Table II** Analysis of deviance from selected regression models analysing cancer of the breast and all other sites reported from 1958 to 1989

| *Terms in model* | *Beast cancer* dev | d.f. | *All other sites* dev | d.f. |
|---|---|---|---|---|
| (a) DEC | 167.39 | 99 | 235.72 | 99 |
| (b) Model A + MO | 129.86 | 88 | 221.01 | 88 |
| (c) DEC + DEC-specific NMO | 108.55 | 90 | 112.91 | 90 |
| (d) Model C + SMO + CMO | 87.175 | 88 | 110.62 | 88 |
| (e) Model C + DEC-specific SMO + DEC-specific CMO | 63.853 | 72 | 97 | 72 |

DEC, decade (period) effects, qualitative; MO, month effects, qualitative; NMO, month effects, quantitative; SMO, sine (month × π/6), quantitative; CMO, cosine (month × π/6), quantitative.

**Table III** Raw parameter estimates with standard error and exponentiated estimates with 95% CI from a simple model with period and month (categorical) effects only

| *Parameter* | *Raw values*[a] *Estimate* | *s.e.* | *Exponentiated values*[b] *Point estimate* | *95% CI* |
|---|---|---|---|---|
| Before 1880 | −7.294 | 0.028 | 0.00 | 0.00–0.00 |
| 1880–1890 | −5.234 | 0.015 | 0.01 | 0.01–0.01 |
| 1890–1900 | −4.355 | 0.012 | 0.01 | 0.01–0.01 |
| 1900–1910 | −3.976 | 0.011 | 0.02 | 0.02–0.02 |
| 1910–1920 | −3.872 | 0.011 | 0.02 | 0.02–0.02 |
| 1920–1930 | −4.002 | 0.012 | 0.02 | 0.02–0.02 |
| 1930–1940 | −4.468 | 0.014 | 0.01 | 0.01–0.01 |
| 1940–1950 | −5.394 | 0.017 | 0.00 | 0.00–0.00 |
| 1950– | −7.877 | 0.034 | 0.00 | 0.00–0.00 |
| January[c] | Reference | | | |
| February | 0.015 | 0.014 | 1.01 | 0.99–1.04 |
| March | −0.010 | 0.014 | 0.99 | 0.96–1.02 |
| April | 0.016 | 0.014 | 1.02 | 0.99–1.05 |
| May | 0.025 | 0.014 | 1.03 | 1.00–1.05 |
| June | 0.048 | 0.014 | 1.05 | 1.02–1.08 |
| July | 0.021 | 0.014 | 1.02 | 0.99–1.05 |
| August | 0.021 | 0.014 | 1.02 | 0.99–1.05 |
| September | −0.008 | 0.014 | 0.99 | 0.97–1.02 |
| October | 0.003 | 0.014 | 1.00 | 0.97–1.03 |
| November | 0.001 | 0.015 | 1.00 | 0.97–1.03 |
| December | −0.022 | 0.014 | 0.98 | 0.95–1.01 |

[a]Parameter estimates are from a model with period and month (categorical) but without an intercept term. [b]Exponentiated values for period represent a crude incidence (number of cases during follow-up period/numbr of live births) for January. Exponentiated values for months represent a relative risk, using January as a reference month. [c]Reference month.

**Table IV** Parameters estimates (and 95% confidence intervals) from a full regression model fitting decade and decade-specific NMO, SMO and CMO to log breast cancer incidence from 1958 to 1989

| *Decade* | *Intercept* | *NMO*[a] | *SMO*[b] | *CMO*[c] |
|---|---|---|---|---|
| Before 1880 | −7.54 (−7.71, −7.37) | 0.04 (0.01, 0.06) | 0.05 (−0.07, 0.16) | 0.01 (0.07, 0.09) |
| 1880–1890 | −5.28 (−5.35, −5.20) | 0.01 (0.00, 0.02) | −0.01 (−0.06, 0.04) | −0.04 (−0.08, −0.01) |
| 1890–1900 | −4.38 (−4.42, −4.33) | 0.00 (0.00, 0.01) | −0.01 (−0.04, 0.03) | −0.02 (−0.04, 0.00) |
| 1900–1910 | −3.96 (−4.00, −3.92) | 0.00 (−0.01, 0.00) | −0.01 (−0.03, 0.02) | −0.02 (−0.04, −0.01) |
| 1910–1920 | −3.85 (−3.89, −3.81) | 0.00 (−0.01, 0.00) | 0.00 (−0.03, 0.03) | −0.03 (−0.05, −0.01) |
| 1920–1930 | −3.95 (−4.00, −3.91) | −0.00 (−0.01, 0.00) | −0.01 (−0.05, 0.02) | −0.01 (−0.03, 0.01) |
| 1930–1940 | −4.40 (−4.46, −4.34) | −0.01 (−0.02, 0.00) | 0.00 (−0.04, 0.05) | 0.01 (−0.01, 0.04) |
| 1940–1950 | −5.33 (−5.42, −5.25) | −0.01 (−0.02, 0.00) | 0.00 (−0.06, 0.06) | −0.02 (−0.06, 0.01) |
| After 1950 | −7.59 (−7.79, −7.39) | −0.04 (0.07, −0.01) | −0.09 (−0.02, 0.05) | 0.14 (0.04, 0.23) |

[a]NMO, month as a continuous variable; [b]SMO, sin (month × π/6.0); [c]CMO, cos (month × π/6.0).

**Figure 1** Observed (—●—) and expected (—□—) natural logarithms of crude breast cancer incidence rates (number of cases/number of live births) from nine different time periods.

seasonality components for breast cancer risk in each decade.

Regression coefficients and 95% confidence intervals for the full model (decade, month as a continuous variable and sine- and cosine-transformed month) for predicting the natural logarithm of breast cancer incidence are presented in Table IV. Figure 1 shows predicted and observed values for the log incidence for all the periods.

### Discussion

This study shows birth seasonality among women who developed breast cancer in Sweden during the period 1958 to 1989. Inclusion of the number of births in the multiplicative model eliminates the possibility that the observed pattern reflects variability in the population at risk. Furthermore, the lack of birth seasonality among Swedish women with cancers at sites other than the breast indicates that differential survival to adult age by season of birth is not a source of bias in our data. Confounding due to exposures later in life is also unlikely, since it is almost inconceivable why they should be associated with month of birth.

Our data cannot be used to examine for the effects of age at diagnosis or cohort effects, since they are confounded. The different birth cohorts were under observation during different age spans during the operation of the Cancer Registry, and were observed for varying periods of time. These two factors are combined in the decade effect (the intercept term in the regression models), and cannot be separated. Despite the fact that the Cancer Registry started operation in 1958, and provided over 30 years of follow-up, we have no birth cohorts that have 100% follow-up between the ages of 30 and 70. Thus, for the earliest birth cohorts, we have only diagnoses among the older women who survived to 1958 (the beginning of follow-up), while for the younger cohorts we have only the patients who developed cancer at younger ages (39 or less for the 1950 and after cohort). Attempts to resolve this confounding by restricting the size of the study base would have seriously reduced the power of the study.

In their statistical appendix, Prener and Carstensen (1990) point out that the analyses using the number of births per month are valid assuming that mortality does not depend on month of birth. An additional unstated assumption is that differences in follow-up time are not dependent on month of birth. Neither of these assumptions was valid for our study, since we observed large, statistically significant interactions of month (NMO) with period. In the oldest cohorts, more patients were born at the end of they year than at the beginning of the year, as evidenced by the positive values of NMO in the regressions with breast cancer (Table IV) or all other sites (data not presented). In the older cohorts risk apparently increases for persons born later in the calendar year, and then suddenly drops at New Year. This finding lacks biological meaning, and we ascribe the increase in risk during the year as an artifact of a differential mortality for those born at the beginning of the year compared with those born at the end of the year. Since persons born in January are, on average, 11 months older than those born in December, more of them have died prior to the start of follow-up, and thus the relative size of the population at risk increases from January to December.

Positive values for NMO in the younger cohorts may be due to varying follow-up times and/or rising age-specific incidence rates. In the last birth cohorts the individuals born in December have been under observation for shorter periods of time and are younger, and thus appear to have a lower risk than the January-born women. Presumably this decrease in incidence for those born in December compared with those born in January would diminish as follow-up time increased and the birth cohorts age. Inclusion of NMO in the models is required to correct for differential mortality and follow-up owing to month of birth in the various birth cohorts.

Prener and Carstensen (1990) achieved reasonable fits to their data without removing the confounding effects of month. However, their study started with younger birth cohorts (the oldest were born in 1900–10), and follow-up started earlier (1943) in the Danish Cancer Registry. The oldest individuals in their study were only 43 years old, thus minimising the effect of differential mortality. In addition, their analyses were done with testes cancer, and the youngest birth cohorts were born in 1950–59. Since the age-specific incidence rates for testes cancer peak at a much younger age, varing lengths of follow-up and increasing age-specific incidence rates were not a problem in their study.

The risk of breast cancer shows a cyclic pattern depending on month of birth. Statistical significance at an arbitrary 95% was achieved only for the periods 1880–90, 1900–10, 1910–20 and after 1950, although the period 1890–1900 was close ($P = 0.90$). Parameter estimates for the cyclic components in periods from 1880 to 1920 were rather similar. They indicate a peak for persons born in the summer months, during May or June. The change in incidence is small, however, corresponding to a fluctuation of approximately 4.8% for the period 1900–1910. Parameter estimates for the period after 1950 were completely different, however, with a maximum in the autumn. These women, at most 39 years old, represent premenopausal breast cancer.

The demonstration of birth seasonality in women with breast cancer born before 1920 represents a powerful argument that perinatal factors influence breast cancer risk. We can only speculate about the nature of such factors, though their effect on breast cancer has clearly diminished. The highest risk is evident in women born in June, the month with the more extensive and intensive daylight. Light reduces melatonin secretion from the pineal gland, and it has been postulated that melatonin suppresses the development of breast cancer (Stevens *et al.*, 1992). However, the melatonin effect has not been established and, if it exists at all, it is more likely to affect tumour progression rather than early, preinitiation, stages. In addition, variation in day length has not changed, though the effect of artificial illumination is not known. Conceivably pregnancy oestrogens, other pregnancy hormones or post-natal exposures (including diet) may have a seasonal variation.

Birth weight may be another risk factor for breast cancer (Ekbom *et al.*, 1992) that shows seasonal variation. During the period 1905 to 1914, children born in Uppsala county during the spring or summer had a higher mean birth weight (3,369 g) than those born in the autumn or winter (3,312 g), possibly because of differences in the diet of the mother during the last trimester. This difference in birth weight, 57 g, has steadily decreased and was only 15 g for the period 1984–86 (3,485 g *vs* 3,470 g).

The principal conclusion of this study is that perinatal factors are important in breast cancer carcinogenesis, although their nature remains elusive. Evidently, such perinatal influences do not directly challenge other hypotheses postulating that genetic or later environmental exposures also contribute to the occurrence of breast cancer.

The authors thank Dr Sven Cnattingius, who made data on the seasonality on birth weight available to us, Professor Reinhold Bergström and Professor Timo Hakulinen for statistical advice and Moa and Emil Ekbom for computerising the monthly birth rates. The study was supported by grants from the Swedish Cancer Society.

### References

ANBAZHAGEN, R. & GUSTERSON, B. (1992). Reversed cerebral asymmetry in women with breast cancer (letter). *Lancet*, **339**, 1056.

ANBAZHAGAN, R., NATHAN, B. & GUSTERSON, B.A. (1992). Prenatal influences and breast cancer (letter). *Lancet*, **340**, 1477–1478.

BERNSTEIN, L., DEPUE, R.H., ROSS, R.K., JUDD, H.L., PIKE, M.C. & HENDERSON, B.E. (1986). Higher maternal levels of free estradiol in first compared to second pregnancy: early gestational differences. *J. Natl Cancer Inst.*, **76**, 1035–1039.

EKBOM, A., TRICHOPOULOS, D., ADAMI, H.-O., HSIEH, C.-C. & LAN, S.J. (1992). Evidence of prenatal influences on breast cancer risk. *Lancet*, **340**, 1015–1018.

HSIEH, C.-C., LAN, S.J., EKBOM, A., PETRIDOU, E., ADAMI, H.-O. & TRICHOPOULOS, D. (1992). Twin membership and breast cancer risk. *Am. J. Epidemiol.*, **136**, 1321–1326.

HSIEH, C.-C., TZONOU, A. & TRICHOPOULOS, D. (1991). Birth order and breast cancer risk. *Cancer Causes and Control*, **2**, 95–98.

MILLER, W.R. (1993). Hormonal factors and risk of breast cancer (editorial). *Lancet*, **341**, 25–26.

PANAGIOTOPOULOU, K., KATSOUYANNI, K., PETRIDOU, E., GARAS, Y., TZONOU, A. & TRICHOPOULOS, D. (1990). Maternal age, parity, and pregnancy estrogens. *Cancer Causes and Control*, **1**, 119–124.

PRENER, A. & CARSTENSEN, B. (1990). Month of birth and testicular cancer risk in Denmark. *Am. J. Epidemiol.*, **131**, 15–19.

SANDSON, T.A., WEN, P.Y. & LEMAY, M. (1992). Reversed cerebral asymmetry in women with breast cancer. *Lancet*, **340**, 1015–1018.

STEVENS, R.G., DAVIS, S., THOMAS, D.B., ANDERSSON, L.E. & WILSON, B.W. (1992). Electric power, pineal function, and the risk of breast cancer. *FASEB J.*, **6**, 853–860.

TAMBY-RAJA, R.L. & RATNAM, S.S. (1981). Plasma steroid changes in twin pregnancies. In *Twin Research*, Vol. 3, Gedda, L., Parisi, P. & Nance, W. (eds) pp. 189–195. Alan R. Liss: New York.

THE CANCER REGISTRY (1960–92). *Cancer Incidence in Sweden 1958–1988*. National Board of Health and Welfare: Stockholm.

THOMPSON, J.A. & JANERICH, D.T. (1990). Maternal age at birth and risk of breast cancer in daughters. *Epidemiology*, **1**, 101–106.

TRICHOPOULOS, D. (1990). Hypothesis: does breast cancer originate *in utero*? *Lancet*, **335**, 939–940.


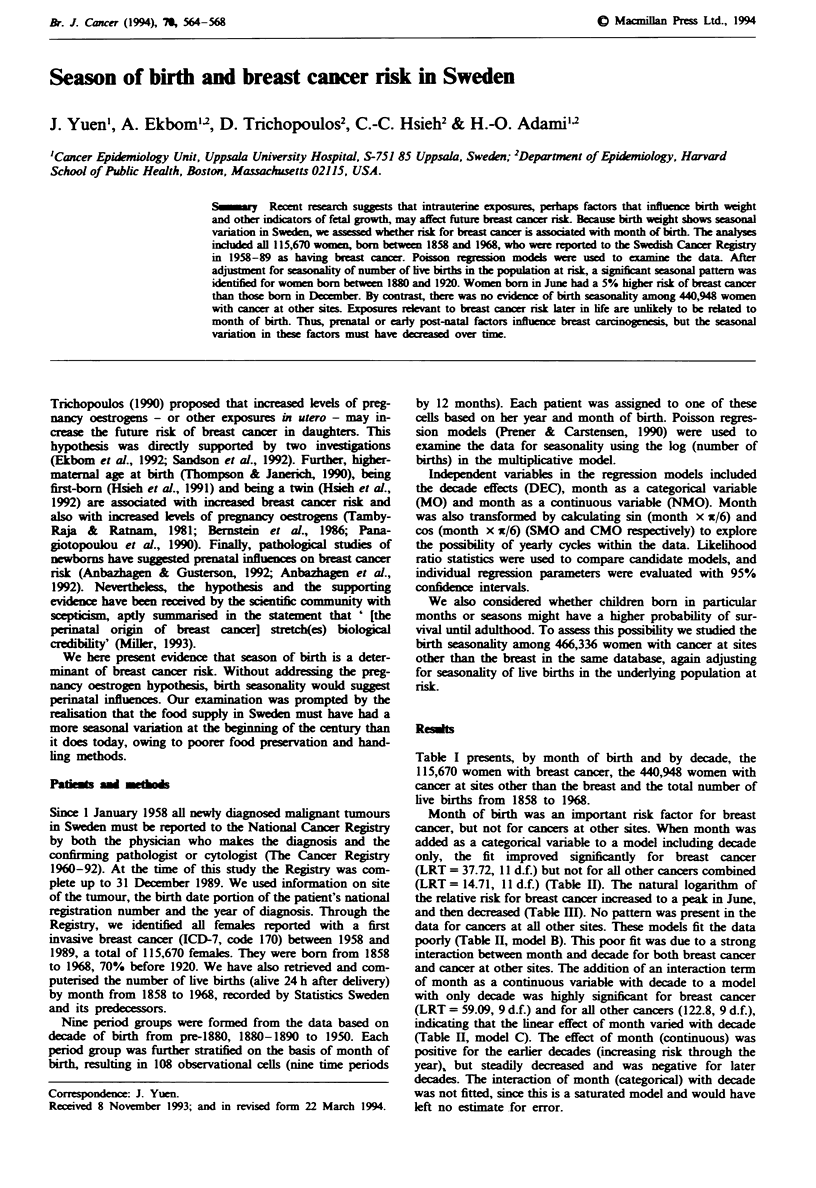

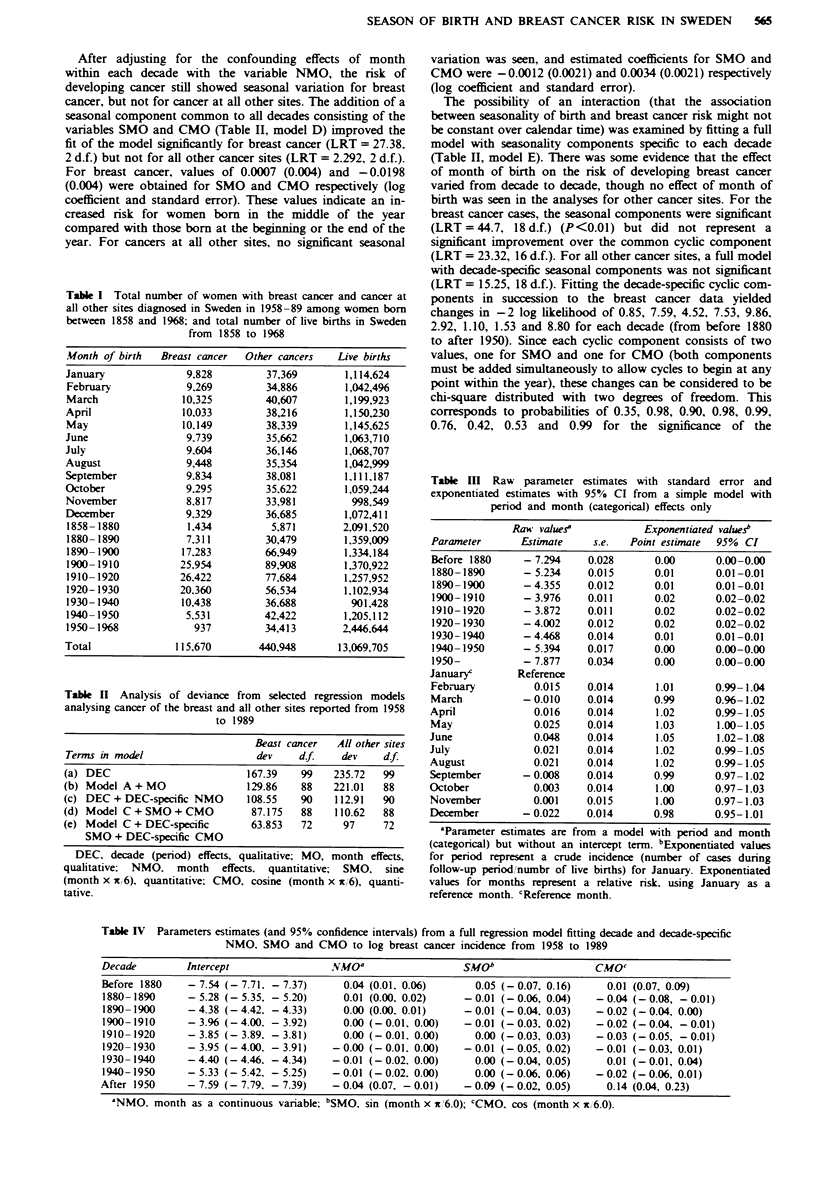

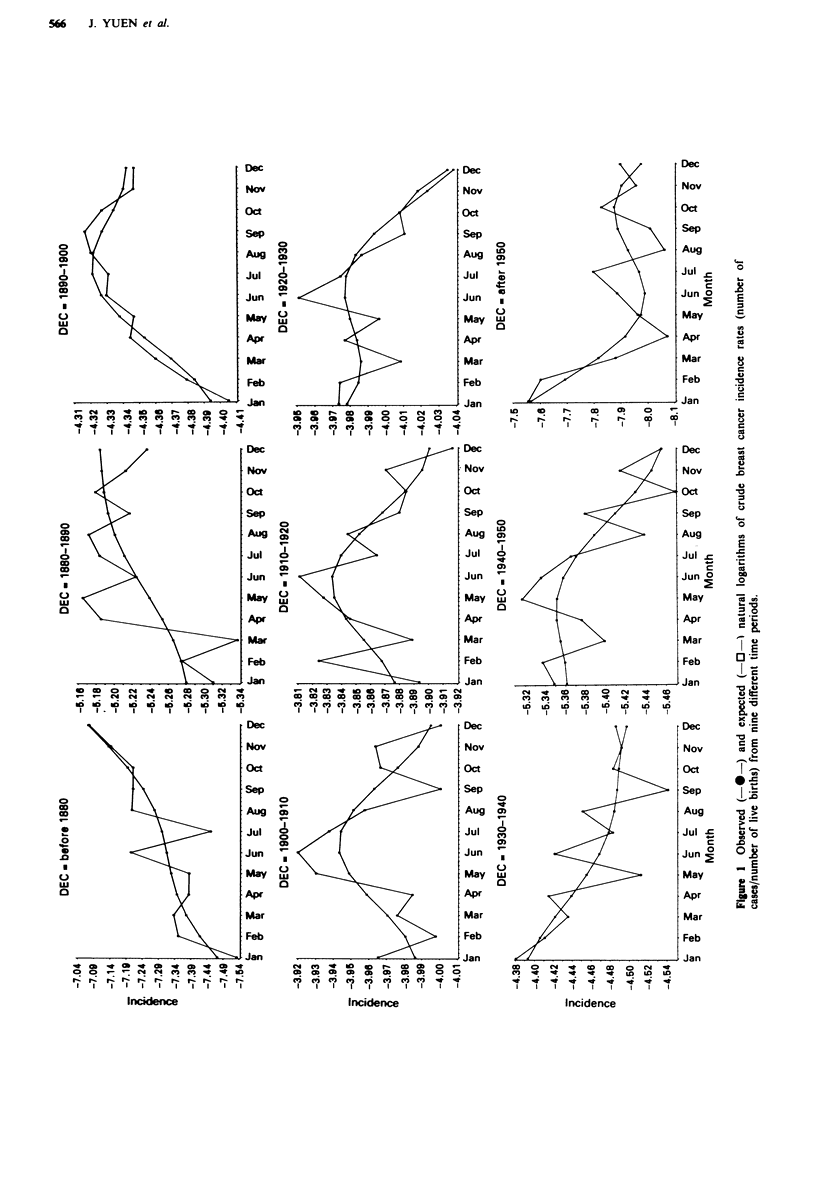

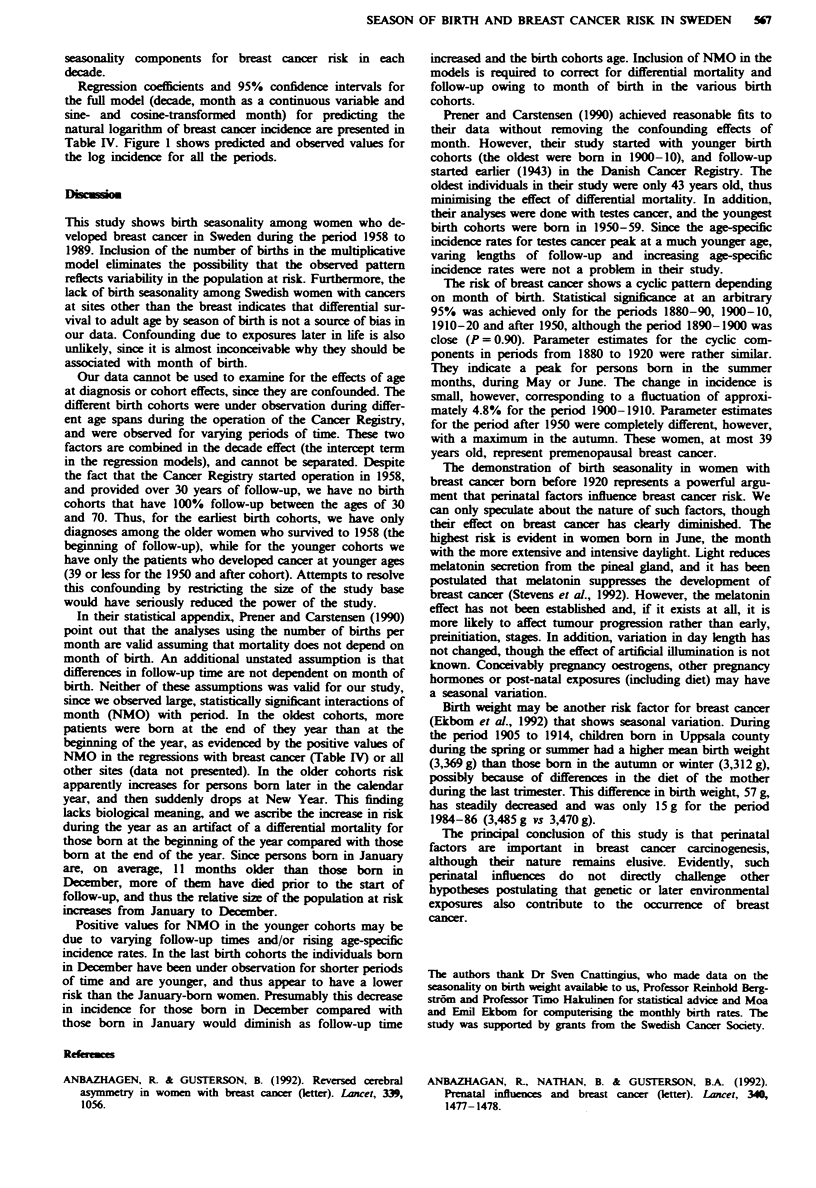

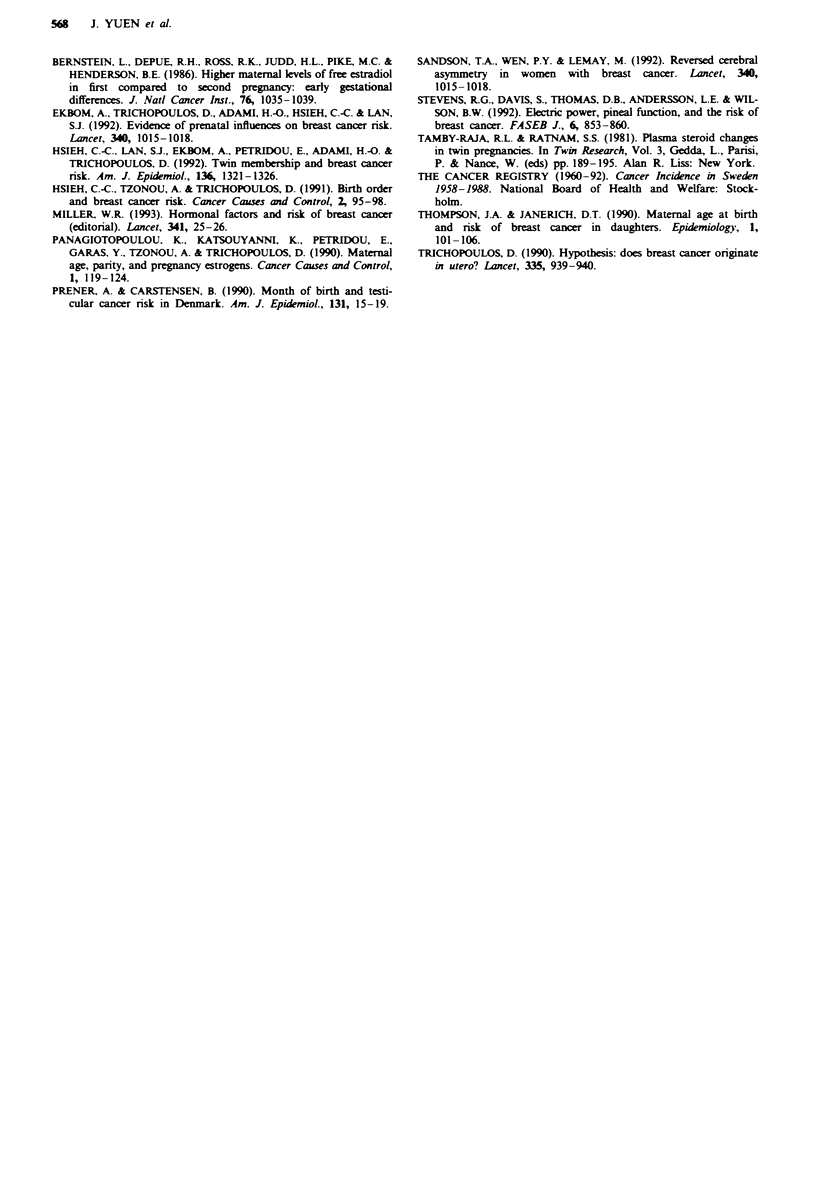


## References

[OCR_00673] Anbazhagan R., Gusterson B. A. (1992). Reversed cerebral asymmetry and breast cancer.. Lancet.

[OCR_00678] Anbazhagan R., Nathan B., Gusterson B. A. (1992). Prenatal influences and breast cancer.. Lancet.

[OCR_00685] Bernstein L., Depue R. H., Ross R. K., Judd H. L., Pike M. C., Henderson B. E. (1986). Higher maternal levels of free estradiol in first compared to second pregnancy: early gestational differences.. J Natl Cancer Inst.

[OCR_00691] Ekbom A., Trichopoulos D., Adami H. O., Hsieh C. C., Lan S. J. (1992). Evidence of prenatal influences on breast cancer risk.. Lancet.

[OCR_00719] Ekbom A., Trichopoulos D., Adami H. O., Hsieh C. C., Lan S. J. (1992). Evidence of prenatal influences on breast cancer risk.. Lancet.

[OCR_00698] Hsieh C. C., Lan S. J., Ekbom A., Petridou E., Adami H. O., Trichopoulos D. (1992). Twin membership and breast cancer risk.. Am J Epidemiol.

[OCR_00701] Hsieh C. C., Tzonou A., Trichopoulos D. (1991). Birth order and breast cancer risk.. Cancer Causes Control.

[OCR_00705] Miller W. R. (1993). Hormonal factors and risk of breast cancer.. Lancet.

[OCR_00709] Panagiotopoulou K., Katsouyanni K., Petridou E., Garas Y., Tzonou A., Trichopoulos D. (1990). Maternal age, parity, and pregnancy estrogens.. Cancer Causes Control.

[OCR_00715] Prener A., Carstensen B. (1990). Month of birth and testicular cancer risk in Denmark.. Am J Epidemiol.

[OCR_00726] Stevens R. G., Davis S., Thomas D. B., Anderson L. E., Wilson B. W. (1992). Electric power, pineal function, and the risk of breast cancer.. FASEB J.

[OCR_00738] Thompson W. D., Janerich D. T. (1990). Maternal age at birth and risk of breast cancer in daughters.. Epidemiology.

[OCR_00743] Trichopoulos D. (1990). Hypothesis: does breast cancer originate in utero?. Lancet.

